# Interplay between AMA1 immunisation and parasite challenge leads to protection against malaria infection in rhesus macaques

**DOI:** 10.1186/1475-2875-9-S2-P14

**Published:** 2010-10-20

**Authors:** Muzamil Mahdi Abdel Hamid, Edmond J Remarque, Bart W Faber, Leonie M van Duivenvoorde, Clemens HM Kocken, Alan W Thomas

**Affiliations:** 1 Institute of Endemic Diseases, University of Khartoum, Sudan; 2 Department of Parasitology, Biomedical Primate Research Centre, Rijswijk, The Netherlands

## Background

**Figure 1:**
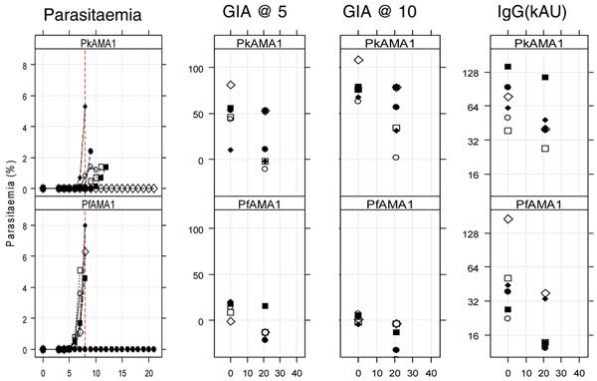


*Plasmodium knowlesi* (Pk), a simian malaria parasite, is a suitable primate model for *Plasmodium falciparum* (Pf), and it was recently identified as the fifth human malaria parasite [1]. To test the ability of yeast-expressed PkAMA1 (Figure 1) to protect rhesus macaques upon challenge with Pk, six healthy rhesus monkeys were vaccinated with PkAMA1 and six control monkeys were vaccinated with PfAMA1 formulated in an oil in water adjuvant [2]. All monkeys received three i.m. vaccinations at 4 week intervals.

## Results

PkAMA1 monkeys produced antibodies that inhibited Pk growth in vitro. Monkeys were challenged two weeks after the third vaccination with Pk blood stage parasites and parasitaemia was followed for 3 weeks. Five out of six controls and one PkAMA1 animal developed fulminent parasitaemias. Four out of six PkAMA1 vaccinated monkeys delayed the onset of the parasitaemia (> 2 days) and one animal in the PkAMA1 group was able to completely control parasitaemia, which correlated with the level of parasite growth inhibitory antibodies. PkAMA1 vaccination delayed the rate of parasite development, but no apparent sterile protection was achieved (Figure 1).

**Figure 2:**
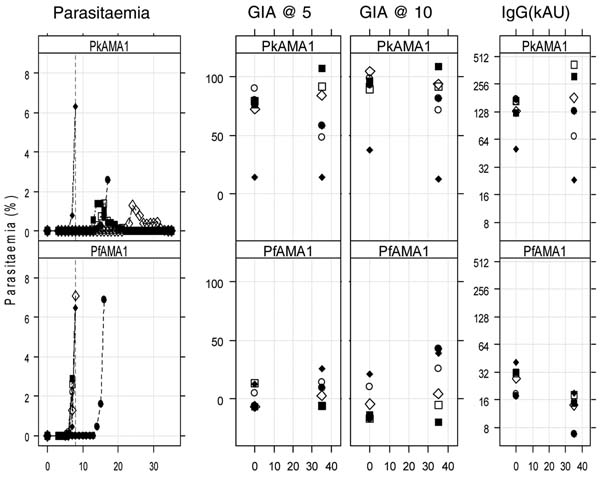


All animals were treated with chloroquine and left to recover, and were subsequently boosted with PkAMA1 or PfAMA1 and challenged two weeks later with Pk blood stage parasites and parasitaemia was followed for 5 weeks. All control animals became parasitaemic and four out of six animals in the PkAMA1 group were able to control the parasitaemia (<1.5%) and subsequently clear the parasites to levels undetectable (Figure 2).

## Conclusion

Protection from Pk challenge is possible albeit non-sterilising. GIA correlates with protection, IgG levels do not at first challenge. Protection improves after challenge-boost. The findings presented here may have implications for Pf challenge models in humans.
